# Passive anterior tibia translation in anterior cruciate ligament-injured, anterior cruciate ligament-reconstructed and healthy knees: a systematic review

**DOI:** 10.1007/s12306-018-0572-6

**Published:** 2018-10-16

**Authors:** M. N. J. Keizer, E. Otten

**Affiliations:** Center for Human Movement Science, University Medical Center Groningen, University of Groningen, Groningen, The Netherlands

**Keywords:** Knee laxity, Influences, ACL, Allograft, Autograft

## Abstract

**Abstract:**

Anterior tibia translation (ATT) is mainly prevented by the anterior cruciate ligament. Passive ATT tests are commonly used to diagnose an anterior cruciate ligament (ACL) injury, to select patients for an ACL reconstruction (ACLR), and as an outcome measure after an ACLR. The aim of this review was to present an overview of possible factors determining ATT. A second purpose was to give a summary of the ATT measured in the literature in healthy, ACL-injured and ACLR knees and a comparison between those groups. A literature search was conducted with PubMed. Inclusion criteria were full-text primary studies published in English between January 2006 and October 2016. Studies included reported ATT in explicit data in healthy as well as ACL-injured or ACLR knees or in ACL-injured as well as ACLR knees. Sixty-one articles met inclusion criteria. Two articles measured the ATT in healthy as well as ACL-injured knees, 51 in ACL-injured as well as in ACLR knees, three in ACLR as well as in healthy knees and three in healthy, ACL-injured and ACLR knees. A difference in ATT is found between healthy, contralateral, ACLR and ACL-injured knees and between chronic and acute ACL injury. Graft choices and intra-articular injuries are factors which could affect the ATT. The mean ATT was lowest to highest in ACLR knees using a bone–patella tendon–bone autograft, ACLR knees using a hamstring autograft, contralateral healthy knees, healthy knees, ACLR knees with an allograft and ACL-injured knees. Factors which could affect the ATT are graft choice, ACL injury or reconstruction, intra-articular injuries and whether an ACL injury is chronic or acute. Comparison of ATT between studies should be taken with caution as a high number of different measurement methods are used. To be able to compare studies, more consistency in measuring devices used should be introduced to measuring ATT. The clinical relevance is that an autograft ACLR might give better results than an allograft ACLR as knee laxity is greater when using an allograft tendon.

**Level of evidence:**

III.

## Introduction

Anterior tibia translation (ATT) is mainly prevented by the anterior cruciate ligament (ACL) [1]. An ACL injury results in higher ATT with respect to the femur. To reduce the increased ATT after an ACL injury, an ACL reconstruction (ACLR) is warranted [2]. Passive ATT tests are commonly used to diagnose an ACL injury and to select patients for an ACLR [3]. Moreover, passive ATT tests are commonly used as an outcome measure after an ACLR, for example, to compare knee laxity after an ACLR using different types of grafts (i.e. [4, 5]).

Several methods can be used to assess the ATT. These tests could either be clinical tests, i.e. the Lachman test, or instrumental measuring methods (i.e. [6, 7]). The most frequently used instrumental measuring method is the KT-1000 arthrometer (KT-1000) (Medmetric Corp., San Diego, CA, USA) developed by Dale Daniel in 1983 [8]. Using the KT-1000 and its successors, the KT-2000 [9] and the ComputKT, an examiner applies forces to the tibia using a handle on top of the device. The anterior–posterior displacement is determined by the distance or relative motion between two sensing paddles: one on the patella and one on the tibial tubercle. The device is calibrated by the determination of the zero point which is done by performing several anterior and posterior translations of the tibia. Visual–manual records are displayed, and audible tones are reached at 15 N, 20 N, 30 N, 67 N, 89 N, 133 N, 134 N, maximal manual (Mm) or maximal personal (Mp) forces. The KT-2000 and the ComputKT have improved data visualisation.

Other methods to assess the ATT are the Kneelax (MR Systems, Haarlem, the Netherlands [10]), the Rolimeter (Aircast, Vista, CA, USA [11]), the Telos Stress Device (H.Tulaszewski, 6302 LICH-Ober-Blessingen, West Germany [12]), the electromagnetic measurement system (EMC) (FASTRAK, Polhemus, VT, USA [13]), the radiostereometric analysis (RSA [12]), fluoroscopic measurements (FM) (BV-29; Philips, Best, the Netherlands) and (computer-assisted) navigation systems. The Kneelax is similar to the KT-1000, but the updated recording process allows digital recording of ATT at the same forces as the KT-1000. The Rolimeter can measure the ATT during the Lachman, anterior drawer and ‘step-off’ tests and is easy in use and cheap. The ends of the device are placed on the mid-patella and tibia, and ATT is measured using a calibrated stylus with 2-mm markers. The Telos Stress Device in combination with a radiostereometric analysis is expensive and results in radiation exposure. When mechanically a force of 150 N, 250 N or maximal manual (Mm) is applied, a stress radiograph is made. Recently, the Telos Stress Device is updated allowing to determine the ATT with a linear optical encoder and without radiographs.

The electromagnetic measurement system (EMS) is an in vivo noninvasive system using an electromagnetic sensor during the pivot-shift test. It monitors instantaneous 3D position and calculates the 3D acceleration of the motion. The radiostereometric analysis (RSA), developed by Selvik et al., has a high accuracy of 0.1-mm displacement. It is an invasive method relying on the implantation of tantalum beads. During a fluoroscopic measurement (FM), the device is placed on the medial side of the knee, and X-ray fluoroscopy captures the knee motion during a Lachman test. A (computer-assisted) navigation system can be used during surgery to measure three-dimensional knee kinematics when applying a specific amount of force on the tibia.

A variety of factors could determine the ATT. It is necessary to identify possible factors which could determine the ATT as knee laxity is shown to be associated with osteoarthritis [14, 15] and an increased chance of knee injuries [16], in particular an ACL injury [17, 18]. Besides, it is not clearly reported what the range of ATT is in healthy, ACL-injured and ACLR knees.

The main purpose of the current systematic review was to give an overview of possible factors determining the ATT. A second purpose was to present a summary of the ATT measured in the available literature in healthy, ACL-injured and ACLR knees and a comparison between those groups.

## Methods

### Inclusion criteria

In order to identify articles for inclusion, a systematic literature search was conducted with the PubMed electronic database on the 6 October 2016. The search terminology was based on the query “(Knee OR ACL) AND (Laxity OR Anterior Translation) NOT (cadaveric OR Shoulder OR Ankle OR PCL OR TKA OR TKR)”.

Titles, abstracts and full texts were analysed by the first author (M.N.J.K). Articles were included if they were: (1) full-text primary studies; (2) published in the English language; (3) published between the 1 January 2006 and 1 October 2016, to reduce the high number of papers and as the measurement methods are improved; (4) studies that reported possible factors determining the ATT; (5) studies that reported ATT in either ACL-injured as well as in ACLR knees; in ACL-injured as well as in healthy knees; or in ACLR as well as in healthy knees; (6) studies that displayed the ATT in explicit data. Review articles were excluded. Articles were excluded when they only measured ATT in ACL-injured, ACLR or healthy participants, did not display ATT in explicit data, measured ATT in participants with additional (knee) injuries, measured tibia position instead of ATT or measured ATT in an active situation.

After identification of the articles, the Newcastle–Ottawa Scale was used by the first author (M.N.J.K.) to appraise the studies that were identified for inclusion in this review [19]. All included studies were found to have an average to good study quality with a score of 6 to 9 out of 9. No reasons were found to assume biases in the data.

### Study characteristics

Articles which met inclusion criteria were analysed for patient demographics, measuring methods to access ATT, the ATT, factors determining the ATT, and, for articles with ACLR participants, type of graft used.

**Fig. 1:**
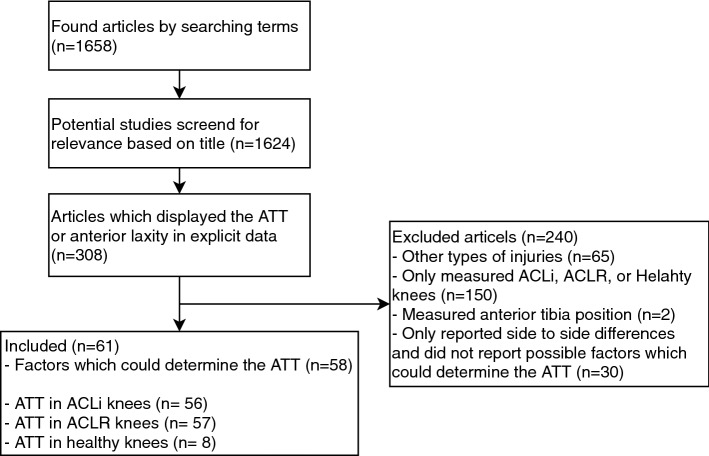
Flow chart of the literature search

Fifty-eight articles reported factors which may determine the ATT. Two articles were identified reporting ATT in ACL-injured as well as healthy or contralateral healthy knees, 51 articles were identified reporting ATT in ACL-injured as well as ACLR knees, and 3 articles were identified reporting ATT in ACLR as well as in healthy knees. Three articles were included in all three groups as they reported ATT in healthy, ACL-injured and ACLR knees. For analyses, sixty-one articles were included (Fig. 1).

The number of included participants per study ranged from 11 to 375. The average age of the participants included per study ranged from 13.9 to 54.4 year. Four studies did not include information on gender. In total, 2.583 of the patients were male, and 1651 were female. Forty-four of the studies used hamstring autografts for ACLR, 15 of the studies used bone–patellar tendon autografts for ACLR, and six of the studies used allograft for ACLR.

### Synthesis of results

The mean ATT was measured for all measurement methods as well as for ACL-injured, ACL-reconstructed (split by type of graft) and (contralateral) healthy knees. These data were compared between groups.

### Statistical analysis

In this review, the results of articles with a significant difference of *p* < 0.05 were declared as significant results.

An independent two-way factorial analysis of variance with interaction was conducted to find the effect of the type of devices and the groups (healthy, contralateral healthy, ACL-injured, ACLR with hamstring autograft tendon, ACLR with bone–patella tendon–bone autograft tendon and ACLR with allograft tendon knees) on the ATT, to find whether there is an interaction between groups and type of devices and to evaluate the coefficients of the groups and devices.

## Results

### Possible factors which could determine the ATT

Chen et al. [20] found that patients which had an acute ACL-injured (*n* = 27) had significantly lower ATT in comparison with patients who had chronic ACL-injured (*n* = 28). Christino et al. [6] found that ATT in patients without intra-articular injuries (*n* = 19) was significantly lower than in patients with intra-articular injuries (*n* = 11).

Of sixteen studies only two articles found significant differences in ATT between using a single-bundle and a double-bundle autograft tendon for ACLR in favour of a double-bundle autograft [4, 21]. Three of the six articles which compared allograft use and autograft use found a significant difference in favour of an autograft tendon [22–24]. Only one out of five studies which compared BPTB autograft and hamstring autograft use reported a significant difference in favour of hamstring autografts [23]. A significantly higher ATT was found in patients who underwent ACLR using a 4-strand compared to an 8-strand hamstring autograft [24], in patients who underwent ACLR using a Leeds-Keio ligament compared to using a BPTB autograft at 2 years after reconstruction [25] and in patients who underwent ACLR using a calcium phosphate-hybridised BPTB autograft in comparison with the conventional method [26].

Two studies reported significant differences between graft fixation methods [27, 28]. However, others did not report any differences in graft fixation methods [21, 29–34]. For all comparisons see Table 1.

**Table 1:** Factors which might determine the anterior tibia translation

Study	Compared	Conclusion
[20]	Acute versus chronic ACL-injured knees	Chronic > acute*
[6]	Before ACLR in patients with versus without intra-articular injuries	With > without*
[35]	Males versus females	Females > males
[36]	Males versus females	Females > males
[37]	Males versus females	Females > males
[4]	SB versus DB hamstring aut	SB > DB*
[38]	SB versus DB hamstring aut	DB > SB
[39]	SB versus DB hamstring aut	SB > DB
[40]	SB versus DB hamstring aut	SB > DB
[41]	SB versus DB hamstring aut	SB > DB
[42]	SB versus DB hamstring aut	DB > SB
[43]	SB versus DB hamstring aut	SB > DB
[44]	SB versus DB hamstring aut	DB > SB
[45]	SB versus DB hamstring aut	0°, 30°, and 90°: SB > DB 60°: DB > SB
[46]	SB versus DB hamstring aut	SB > DB
[47]	SB versus DB hamstring aut	DB > SB
[48]	SB versus DB hamstring aut	SB > DB
[49]	SB versus DB hamstring aut	DB > SB
[50]	SB versus DB hamstring aut	SB > DB
[51]	SB versus DB BPTB all	SB > DB
[52]	TB versus SB hamstring aut	KT-1000: TB > SB Telos: SB > TB
[19]	Anatomic versus nonanatomic DB hamstring	SB > anatomic* Nonanatomic > anatomic
[53]	All versus hamstring aut	All > aut
[28]	All versus BPTB aut	All > aut
[54]	All versus hamstring aut	All > aut*
[55]	Hamstring aut versus irradiated all	All > aut*
[22]	BPTB aut versus fresh-frozen all (all1) or y-irradiated all (all2)	All2* > all1 > aut
[56]	All free tendon Achilles versus hamstring aut	All > aut
[23]	BPTB versus hamstring aut	BPTB > hamstring*
[57]	BPTB versus hamstring aut	Hamstring > BPTB
[58]	BPTB versus hamstring aut	Hamstring > BPTB
[59]	BPTB versus hamstring aut	Hamstring > BPTB
[60]	DB hamstring (1) versus BPTB (2) versus BPTB_L (3)	Medial: 3 > 2 > 1 Lateral: 2 > 3 > 1 BPTB_L reduced most*
[61]	DB hamstring aut versus aug	KT-1000: DB > aug Telos: aug > DB
[62]	4-Strand versus 8-strand hamstring aut	4-strand > 8-strand
[63]	Hamstring versus quadriceps aut	Quadriceps > hamstring
[25]	BPTB versus LK	2 y after ACLR: LK > BPTB* 5 y after ACLR: BPTB > LK
[64]	Qf versus BPTB	BPTB > Qf
[65]	Cas versus non-Cas surgery	Non-Cas > Cas
[66]	High versus low tension BPTB or hamstring aut	High > low
[26]	CaP versus CM BPTB	CM > CaP*
[67]	A20 versus P20 versus A20P0 versus A20P20 versus A20P45 bundle fixation	P20 > A20* A20 > A20P0* P20 > A20 > A20P20* P20 > A20 > A20P45*
[68]	With versus without navigation system	With > without
[21]	TT versus AM SB hamstring aut	TT > AM
[69]	Metal versus PLLA screw	Metal = PLLA
[70]	BioCryl versus RigidFix fixation	BioCryl > RigidFix
[27]	Cortical with versus without aperture fixation	Without > with*
[29]	TransFix versus Endobutton fixation	Endobutton > TransFix
[31]	TransFix versus bioscrew fixation	Bioscrew > TransFix
[71]	Bioabsorbable versus metal screw fixation	Metal > bioabsorbable
[72]	Metal versus PLLA screw hamstring aut	PLLA > metal
[73]	RigidFix and intrafix (1) versus RigidFix and bioscrew (2) versus bioscrew and intrafix (3) versus bioscrew and bioscrew fixation (4)	3 > 2 > 4 > 1
[74]	Femoral knot/press fit (1) versus femoral interference screw fixation (2)	2 > 1
[75]	Early extension versus late extension during rehabilitation	Late > early
[76]	Greater than 20% versus lower than 20% strength deficit	Greater > lower
[77]	Three-day versus 2-week immobilisation	3 Days > 2 weeks
[78]	Brace versus nonbrace after ACLR	Nonbrace > brace
[34]	Left-handed versus right-handed physiotherapists using the KT-1000	LH > RH*

*ACL* anterior cruciate ligament, *ACLR* anterior cruciate ligament reconstruction *SB* single bundle, *DB* double bundle, *all* allograft, *aut* autograft, *BPTB* bone–patellar tendon–bone, *TB* triple bundle, *BPTB-L* mono-bundle BPTB combined with extra-articular reconstruction, *aug* remnant-preserving augmentation, *LK* Leeds-Keio ligament, *y* years, *Qf* quadruple flexor, *Cas* computer-assisted surgery, *A20* anteromedial bundle fixation only at 20° of flexion, *P20* posterolateral bundle fixation only at 20° of flexion, *A20P0* anteromedial bundle fixation at 20° and posterolateral bundle fixation at 0° of flexion, *A20P20* anteromedial bundle fixation at 20° and posterolateral bundle fixation at 20° of flexion, *A20P45* anteromedial bundle fixation at 20° and posterolateral bundle fixation at 45° of flexion, *TT* transtibial femoral tunnel preparation, *AM* anteromedial femoral tunnel preparation, *CaP* hybridising calcium phosphate, *CM* conventional method, *PLLA* biodegradable interference screw, *LH* left-handed, *RH* right-handed

*Significant

### Factor analysis of groups and devices on ATT

Two devices (FM and EMS) showed much higher ATT than the other devices, and therefore, these two devices were excluded for calculation of mean ATT per group. A nonsignificant interaction between groups and devices was seen (*p* = 0.73). No *p* values could be calculated for the groups and devices separately, as the number of observations of some groups and some devices was too low. The coefficients of all groups ranged from − 1.75 to 2.89 with a mean of 0.00. The coefficients of all devices ranged from − 3.30 to 4.07 with a mean of 0.21. In Table 2 the coefficients of the groups, of devices which were lower than − 1 and higher than 1, and of the interaction which were lower than − 3 and higher than 3 are reported.

**Table 2:** Highest coefficients of an independent two-way factorial analysis of variance with interaction. Only coefficients for devices lower than − 1 and higher than 1 are reported. Only interaction coefficients lower than − 3 and higher than 3 are reported

Group	Coef	Device	Coef	Device	Coef	Interaction	Coef
ACL-injured	2.89	ComputKT (134 N)	4.07	KT-1000 (133 N)	− 1.26	Telos * healthy	4.51
Contralateral	0.85	Navigation	3.59	Kneelax (98 N)	− 1.45	Telos (150 N) * ACL-injured	4.17
Allograft	− 0.18	Navigation (MF)	2.64	KT-1000 (15 N)	− 1.84	Rolimeter (Mm) * contralateral	3.82
Healthy	− 0.24	KT-1000 (Mm)	1.84	Telos (150 N)	− 2.56	KT-1000 (89 N) * hamstring	3.58
BPTB	− 1.53	KT-1000 (300 N)	1.68	Kneelax (132 N)	− 2.79	Navigation (100 N) * ACL-injured	3.24
Hamstring	− 1.75	Rolimeter	1.66	Rolimeter (Mm)	− 3.30	RSA * BPTB	3.02
		KT-1000 (134 N)	1.02			ComputKT (134 N) * ACL-injured	− 3.16
		KT-2000 (Mm)	1.01			KT-1000 (Mp) * ACL-injured	− 3.38

*Coef* coefficient

**Fig. 2:**
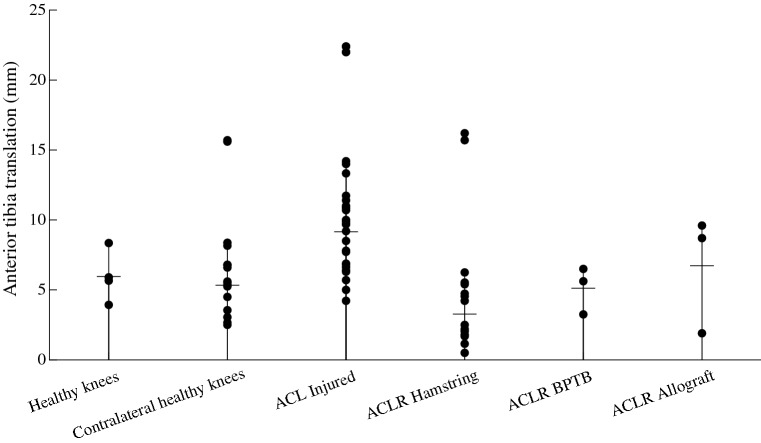
Absolute anterior tibia translation per group (healthy, contralateral healthy, ACL injured, ACL reconstructed with hamstring autograft tendon, ACL reconstructed with bone–patella tendon–bone autograft tendon and ACL reconstructed with allograft tendon knees) of each device (black dots). The black horizontal lines indicate the mean ATT of the groups. The six separate dots indicate the devices excluded from analysis

For the ATT for each device per group see Fig. 2.

## Discussion

The mean finding of this review was that graft choice, ACL injury or reconstruction, intra-articular injuries and whether an ACL injury is chronic or acute are factors which could determine the ATT. Other possible factors, such as fixation techniques, were inconclusive. The mean absolute ATT is, respectively, lowest to highest in ACLR knees with a BPTB autograft (3.25 mm), ACLR knees with a hamstring autograft (3.27 mm), contralateral healthy knees (5.33 mm), healthy knees (5.96 mm), ACLR knees with an allograft tendon (6.73 mm) and ACL-injured knees (9.15 mm).

The mean ATT measured in ACLR knees with an allograft was twice as high as the ATT measured in ACLR knees with an BPTB autograft. This finding is consistent with the finding of Tian et al. [55], however, in contrast with the finding of Ghodadra et al. [28] who did not report a significant difference between BPTB autograft and allograft use. In addition, Laoruengthana et al. [23] reported that ATT is significantly higher in ACLR knees with BPTB autograft compared to ACLR knees with hamstring autograft. This is in contrast with the data presented in this review. The mean ATT in knees with a BPTB autograft was only 0.02 mm lower than in ACLR knees with a hamstring autograft.

The methods used to assess ATT might have introduced a systematic measurement error. In healthy knees, ATT ranged from 3.93 mm (KT-1000 89N) to 8.35 mm (ComputKT) and in contralateral healthy knees from 2.5 mm (KT-1000 67N) to 15.7 mm (EMS). In addition, the range of coefficients measured using an independent two-way factorial analysis of variance with interaction was greater in range for the devices (range: − 3.30 to 4.07) in comparison with the groups (range: − 1.75 to 2.89). Therefore, comparison of ATT between devices should be taken with caution as the choice of measuring device might be paramount. However, some interactions between devices and groups were strong (Table 2), for example: the coefficient of the interaction between the ComputKT and BPTB group was − 3.16. Therefore, the difference in ATT between devices might also have been caused by differences in characteristic of subjects measured in the studies in which those devices were used. More consistency in measuring device used to assess ATT should be introduced. Pugh et al. [30] in their review suggest that the KT-1000 and the Rolimeter provide better results than the Telos Stress Radiography and some other devices not covered in this review. Fortunately, the KT-1000 arthrometer is the most frequently used.

For almost all devices a variety of forces can be used to measure the ATT. The relation between forces and ATT is reported by Lin et al. [33] for healthy knees and ACL-injured knees. They reported a significantly larger displacement and a significantly larger stiffness of the injured knee compared to healthy knees. In line with these results the mean ATT of the studies in this review was smaller in healthy and contralateral knees in comparison with ACL-injured knees. However, the relationship between force and ATT is not seen in the data of the current review. This might be due to differences between studies in, i.e. subject characteristics and other factors which could also have determined the ATT. For example, a significant difference in ATT measured using a KT-1000 between right-handed physiotherapists and left-handed physiotherapists was reported by Sernert et al. [34]. A between-studies comparison of ATT should be taken with caution, especially when different measurement methods or forces are used.

Muscle activity might also have determined the ATT. Klyne et al. [79] found in patients with an ACL injury a relation between ATT in a passive situation and prolonged muscle activity of the medial gastrocnemius during a jump test. Barcellona et al. [80] found that hamstrings activity reduces anterior knee laxity in a passive situation in ACL-injured patients. This indicates that patients with an ACL injury might compensate for knee laxity by increasing the duration of muscle activity. However, Goradia et al. [76] did not find a difference in ATT between patients with strength deficit and patients without strength deficit. Kvist [81] found that there is no correlation between ATT in a passive situation and ATT in an active situation, which might indicate that muscle activity does play a role in the control of ATT during activity. Future studies could investigate the effect of muscle activity on ATT and could investigate ATT in an active situation, i.e. by using the method to assess ATT of Boeth et al. [82].

Some limitations of this review should be addressed. Systematic reviews are limited by the weaknesses of each study. This might include a small number of participants, a short-term follow-up time and a high variability of participants. However, no reasons were found to assume biases in the data. One article which was determined to have a poor quality was excluded from further analysis. In addition, a limitation of this review was the large variety of measurement devices used to assess the ATT, which made a comparison between studies difficult. However, this also makes clear that more consistency should be introduced in measuring method for ATT.

## Conclusion

Surprisingly reported ATT, in comparison with healthy knees, is higher after an ACLR using an allograft tendon and lower in knees using a bone–patella tendon–bone autograft. In addition, ATT was significantly higher in chronic than in acute ACL injuries and in knees with intra-articular injuries compared to knees without intra-articular injuries. Inconclusive results were found for other factors such as fixation techniques. When excluding two devices which measured much higher ATT than the other devices, mean ATT was lowest to highest in ACLR knees using a bone–patella tendon–bone (BPTB) autograft, ACLR knees using a hamstring autograft, contralateral healthy knees, healthy knees, ACLR knees with an allograft and ACL-injured knees. Between-studies comparison of the ATT should be taken with caution as lots of different measurement methods with different forces were used to measure the ATT. To make the compatibility of studies more reliable, more consistency in measuring methods to assess ATT should be introduced. The clinical relevance of this study is that even though the ATT was smaller after an ACLR in comparison with ACL-injured knees, using an allograft tendon, the ATT was greater than healthy knees, whereas by using an autograft tendon the ATT was smaller than in healthy knees. An increase in ATT is found to be a risk factor for osteoarthritis and a chance of knee injuries; therefore, an autograft ACLR might give better results in comparison with an allograft ACLR.
